# Redox Components: Key Regulators of Epigenetic Modifications in Plants

**DOI:** 10.3390/ijms21041419

**Published:** 2020-02-19

**Authors:** Saravana Kumar R. M., Yibin Wang, Xiaopan Zhang, Hui Cheng, Lirong Sun, Shibin He, Fushun Hao

**Affiliations:** State Key Laboratory of Cotton Biology, Key Laboratory of Plant Stress Biology, School of Life Sciences, Henan University, Kaifeng 475004, China; saravana80@126.com (S.K.R.M.); 18238213127@163.com (Y.W.); xiaopanzhan@163.com (X.Z.); 15194627687@163.com (H.C.); sunlr9208@henu.edu.cn (L.S.)

**Keywords:** epigenetic modifications, DNA methylation, histone modification, chromatin remodeling, redox regulation, reactive oxygen species, nitric oxide, antioxidants

## Abstract

Epigenetic modifications including DNA methylation, histone modifications, and chromatin remodeling are crucial regulators of chromatin architecture and gene expression in plants. Their dynamics are significantly influenced by oxidants, such as reactive oxygen species (ROS) and nitric oxide (NO), and antioxidants, like pyridine nucleotides and glutathione in plants. These redox intermediates regulate the activities and expression of many enzymes involved in DNA methylation, histone methylation and acetylation, and chromatin remodeling, consequently controlling plant growth and development, and responses to diverse environmental stresses. In recent years, much progress has been made in understanding the functional mechanisms of epigenetic modifications and the roles of redox mediators in controlling gene expression in plants. However, the integrated view of the mechanisms for redox regulation of the epigenetic marks is limited. In this review, we summarize recent advances on the roles and mechanisms of redox components in regulating multiple epigenetic modifications, with a focus of the functions of ROS, NO, and multiple antioxidants in plants.

## 1. Introduction

Epigenetic modifications refer to the mitotically- or meiotically-inheritable changes in gene expression that are not affected by the DNA sequence itself, mainly including DNA methylation, histone modifications, chromatin remodeling, and histone variants in plants and other organisms [1,2]. They can change chromatin architecture, affect DNA accessibility, and gene activity, thereby regulating many molecular processes, like the transcription of genes, and replication, repair, and recombination of DNA [1,2,3,4]. They play vital roles in controlling growth and development, including cell differentiation, regeneration, reproduction, flowering, and senescence, and governing plant acclimations to various environmental stimuli, such as pathogen infection, drought, high salinity, extreme temperature, heavy metal stresses [1,3,4,5,6,7]. Most epigenetic modifications are reversible, and under the control of multiple factors including different developmental cues, diverse environmental stresses, phytohormone signals [1,8,9]. Among these, redox components are of great importance [10,11,12].

Redox components consist of numerous oxidants and antioxidants. In plants, the primary oxidants are reactive oxygen species (ROS), for example, hydrogen peroxide (H_2_O_2_), superoxide radical (O_2_^•−^), singlet oxygen (^1^O_2_), and hydroxyl radicals, and reactive nitrogen species, including nitric oxide (NO), peroxynitrite, nitrogen dioxide radicals. [13]. The main antioxidants include enzymatic antioxidants (e.g., superoxide dismutase (SOD), catalase (CAT), ascorbate peroxidase (APX), glutathione peroxidase (GPX), glutathione reductase (GR)), and nonenzymatic antioxidants (e.g., pyridine nucleotides (NAD(P)H), glutathione (GSH), glutaredoxin (GRX), ascorbate (ASC), and nicotinamide) [14]. These oxidants and antioxidants are able to spatiotemporally change the redox status and influence redox balance, controlling nearly every aspect of cellular processes such as gene expression, biological metabolisms, growth and development, and adaptations to different environmental stresses in plants [13,15,16,17,18].

In recent years, many review papers covering the roles and regulatory mechanisms of epigenetic modifications have been published. The relationship between redox metabolites and some epigenetic modifications has also been discussed [8,10,11,12,19,20,21,22]. However, the functions and mechanisms of redox mediators modulating the epigenetic modifications are not comprehensively summarized. In this review, we provide an integrated view how redox components control the epigenetic marks, with a focus of the roles of ROS, NO, and multiple antioxidants in the regulation of DNA methylation, histone methylation, and histone acetylation in plants.

## 2. Epigenetic Modifications in Plants

### 2.1. DNA Methylation

DNA methylation typically means the specific post-replication modification over DNA molecules, in which some cytosine bases are methylated at 5′ position to become 5-methyl-cytosine (^5m^C). In plants, methylation occurs in the C base of “CG”, “CHG”, and “CHH” (H represents A, C, or T) contexts [23]. DNA methylation favors the maintenance of genome stability, suppresses gene recombination and mutation, and is essential for the silencing of transposable elements and the regulation of gene expression and splicing [3,24]. DNA methylation inhibits transcriptional initiation, and may have little effect on transcriptional elongation within the gene body [25].

In *Arabidopsis thaliana*, de novo DNA methylation is established through RNA-directed DNA methylation (RdDM) pathway, which involves 21-, 22-, and 24- nt small interfering RNAs (siRNAs) production [3,26,27]. DNA methylation is maintained through three pathways: DNA methyltransferase 1 (MET1) for CG methylation, chromomethylase 3 (CMT3) and CMT2 for CHG methylation, and domain rearranged methyltransferase 2 (DRM2), CMT2, and CMT3 for CHH methylation. The methyl donor is S-adenosyl-l-methionine (SAM) [3,23]. Methyl groups can be removed from DNA through DNA demethylation. Active DNA demethylation is mediated by 5-methylcytosine DNA glycosylases through a DNA base excision repair pathway in plants [3,28]. There exist four 5-methylcytosine DNA glycosylases in *A. thaliana*: repressor of silencing 1 (ROS1), demeter (DME), and Demeter-like 2 (DML2) and DML3 [3,28].

### 2.2. Histone Methylation and Acetylation

Chromatin is the organized nucleoprotein structure in nuclei where nucleosomes are arranged. Each nucleosome is comprised of two copies of H2A, H2B, H3, and H4 histone molecules, and is wrapped by 145~147 bp double-stranded DNA [29]. The N-terminal tails of histones are subject to various post-translational modifications such as methylation, acetylation, phosphorylation, ubiquitinylation, glycosylation, ADP-ribosylation, and sumoylation. These modifications can alter chromatin structure and gene transcription either by affecting the interaction between histones and the surrounding DNA or by modulating the binding of various regulatory proteins to DNA [1,2,7]. Histone methylation and acetylation have been well characterized. They have wide functions in plant evolution, development, and stress acclimations by facilitating or repressing gene expression [22,30,31,32].

Histone methylation is confined to lysine and arginine residues located at different positions of histone molecules (H3, H4). The transfer of methyls to histone is catalyzed by histone methyltransferase (HMT) families, which include histone lysine methyltransferases (HKMTs) and protein arginine methyltransferases (PRMTs) [22,33]. The donor of methyl groups for histone methylation is also SAM. On the basis of the number of methyls that occurs over histone molecules, histone methylation can be grouped into mono-, di-, and tri-methylation. Different modifications have distinct effects on gene expression [22,33]. For instance, trimethylation of Lys 27 (H3K27me3) leads to the repression of gene expression whereas trimethylation at Lys 4 (H3K4me3) activates gene transcription in *A. thaliana* [34,35]. Methyl groups can be removed by histone demethylases (HDMs). In plants, HDMs are grouped into lysine-specific demethylase 1 (LSD1) and Jumonji C domain-containing proteins (JMJs). Both enzymes follow different pathways to demethylate histones using different cofactors. LSD1 pertains to flavin-dependent amine oxidase family whereas JMJs belong to 2-oxoglutarate-dependent dioxygenase family [33,36].

Histone acetylation is the covalent modification in which acetyl groups are transferred from acetyl CoA to the epsilon-amino group of the lysine residue in histone molecules. Such modification causes the neutralization of the positive charge of the lysine, weakens the interaction between the modified histone and DNA, thus, chromatin becomes relaxed [22,37]. Acetylated histones can also recruit other proteins, which regulate chromatin structure [38,39]. Generally, hyperacetylation of histones favors transcriptional activation whereas hypoacetylation of histones causes gene repression [22,37,40]. The levels of histone acetylation are regulated by the antagonistic activities of histone acetyltransferases (HATs) and histone deacetylases (HDACs) [1,41]. Plant HATs are divided into four classes, including p300/CREB (cAMP responsive element-binding protein)-binding proteins, TATA-binding protein-associated factors, general control nonrepressible 5-related N-terminal acetyltransferases and MOZ, Ybf2/Sas3, Sas2, and Tip60 proteins [41]. HDACs in plants are grouped into three types: reduced potassium dependency 3/histone deacetylase 1 (RDP3/HDA1), silent information regulator 2 (SIR2), and plant-specific histone deacetylase 2 (HD2). SIR2 family proteins (sirtuins) require NAD^+^ as cofactor, and other HDACs use Zn or Fe ion as cofactors. There exist 18 HDAC members in *A. thaliana* [41,42].

### 2.3. Chromatin Remodeling

The regulated change of chromatin structure is termed as chromatin remodeling. It can be changed not only by covalent modifications of histones and DNA, but also by ATP-dependent chromatin remodelers and other chromatin-associated factors. ATP-dependent chromatin remodelers are able to cause the alteration of nucleosome position, destabilization of nucleosomes or displacement of canonical histones by histone variants [43]. Eukaryotic ATP-dependent chromatin remodelers are evolutionarily conserved protein complexes that typically possess a catalytic core: ATPase/helicase of the switching defective2/sucrose non-fermenting2 (SWI2/SNF2) family. They perform functions using the energy provided by ATP hydrolysis [43,44]. In plants, ATP-dependent chromatin remodelers are divided into four major subfamilies, including SWI/SNF subfamily, imitation switch subfamily, chromodomain helicase DNA-binding (CHD) subfamily, and inositol requiring 80/SWI2-related ATPase 1 subfamily [43,44].

## 3. Redox Components

### 3.1. ROS and NO

ROS are byproducts of the aerobic metabolism. They act as crucial signaling molecules to mediate and integrate various growth and environmental signals to control plant development, stomatal movement, and acclimation to diverse biotic and abiotic stresses [13,18,45,46]. ROS are generated in distinct organelles or subcellular compartments like chloroplasts, mitochondria, peroxisomes, and apoplasts under normal, especially stressful conditions [18]. Photosynthesis, photorespiration, and mitochondrial electron transport are major sources of ROS. Plasma membrane NADPH oxidase, also known as respiratory burst oxidase (RBOH), is also a key producer of ROS. RBOH-dependent ROS have been demonstrated to be essential regulators of many cellular processes such as seed germination, root formation, tip growth, flowering, stomatal movement, and adaptations to different environmental stimuli in plants [47,48,49,50,51,52]. Under normal conditions, the concentrations of ROS in tissues are relatively low, and ROS can act as signal molecules. However, under stresses, ROS accumulation in plants increases. When the levels of ROS exceed certain thresholds, oxidative stress occurs. High levels of ROS damage various biological molecules and cell structure, even causing cell death in plants [18,53].

Similar to that of ROS, production of NO is an inevitable process in plant metabolism. NO is synthesized in different compartments of cells including cytosol, chloroplasts, mitochondria, and peroxisomes [54,55]. NO reductases, nitrate reductases, and nitric oxide synthase-like enzyme have been addressed to be important sources of NO in plants. NO is also a key secondary messenger. It independently or synergistically acts with ROS to regulate a wide range of cellular events including vegetative growth, reproductive development, stomatal opening and closure, and responses to diverse biotic and abiotic stresses [16,20,56]. NO and H_2_O_2_ can easily enter the nucleus through nuclear pores, and react with nuclear proteins, including histones and transcription factors in plants [15,57,58].

### 3.2. Enzymatic and Nonenzymatic Antioxidants

Cellular redox status constantly undergoes fluctuations that are balanced by different oxidant and antioxidant systems during plant development and in response to stress cues. Of the enzymatic antioxidants, SOD catalyzes the dismutation of O_2_^•−^ into O_2_ and H_2_O_2_. CAT, APX, and GPX convert H_2_O_2_ into O_2_ and H_2_O, whereas GR is responsible for the conversion of the oxidized glutathione to the reduced one [59,60]. Of the non-enzymatic antioxidants, NAD(P)H, GSH, and ASC are critical soluble redox carrier molecules. They can interchange between the oxidized and reduced states (NAD(P)^+^/NAD(P)H, GSSG/GSH, and Asc/DAsc). Their capacity to gain or lose electrons also makes them versatile carriers to alter the activities of many enzymes implicated in numerous important metabolic pathways and cellular events. NAD(P)H is vital for the transmission of redox signals. By supplying reducing equivalents to GSH and ASC through the Asada–Foyer–Halliwell cycle [61], NAD(P)H can process ROS and reactive nitrogen species [60]. GSH and ASC directly interact with H_2_O_2_, and catalyze the conversion of H_2_O_2_ to H_2_O and O_2_. Under oxidative conditions, GSH-GSSG equilibrium shifts towards oxidized glutathione, leading to S-glutathionylation of proteins, an important redox-modification in plants [60,62,63].

## 4. Redox Regulation of Epigenetic Modifications

### 4.1. Redox Regulation of DNA Methylation

Accumulating evidence indicates that redox intermediates govern DNA methylation levels and gene transcription in plants. In general, increases in ROS accumulation cause DNA hypomethylation. In tobacco, addition of O_2_^•−^ inducer paraquat increases the oxidative stress of cells. The expression of *Glycerophosphodiesterase-Like* (*NtGPDL*) is upregulated, and CG sites in the coding regions of *NtGPDL* are selectively demethylated [64]. Similarly, treatment of tobacco suspension cells with jugalone, a toxic plant secondary metabolite 5-hydroxy-1,4-naphthoquinone, increases the ROS levels in nucleus, nucleolus, cytoplasm, and plasma membrane, accompanied with DNA hypomethylation and programmed cell death (PCD) [65]. Additionally, application of 2,2’-azobis (2-amidinopropane) dihydrochloride, a generator of free radicals, in *Pisum* *sativum* suspension culture clearly decreases the global DNA methylation levels [66].

Similar to ROS, NO negatively modulates DNA methylation in plants. For instance, treatment of rice seedlings with high concentrations of sodium nitroprusside (SNP, a NO donor) leads to DNA hypomethylation predominantly at the CHG sites, and growth inhibition. In addition, the NO-evoked alterations in DNA methylation can be inherited to the next generation [67]. In another study, application of low concentration of SNP to heat-treated *Lablab purpureus* plants causes the reduction in the levels of O_2_^•−^ and H_2_O_2_ and the alteration of DNA demethylation and methylation levels [68]. Additionally, antioxidant nicotinamide, an essential component of NAD(P)H, has shown to induce DNA hypomethylation in *P. sativum* [66].

**Figure 1:**
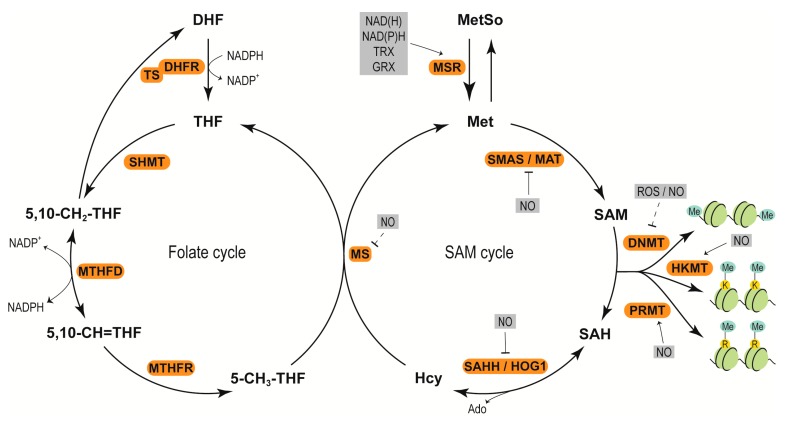
Redox components modulate SAM synthesis through folate cycle in plants. Folate cycle begins with the conversion of DHF to THF through DHFR by utilizing the reducing equivalents from NADPH. Methyls derived from THFs (5,10-CH2-THF, 5,10-CH=THF) are synthesized by SHMT and MTHFD, respectively. 5,10-CH=THF is reduced to 5-CH3-THF by MTHFR. The methyl group from 5-CH3-THF is transferred to Hcy to synthesize Met through MS. The produced Met generates SAM through SAMS. SAM donates methyl groups to DNA or proteins through DNA methyltransferase (DNMT)/HKMT/PRMT, and gets converted to SAH. SAH is further processed to Hcy through SAHH/HOG1. The key enzymes influenced by the cellular redox components are: SAMS/MAT, DNMT/HKMT/PRMT, SAHH/HOG1, MS and MSR. K: lysine; R: arginine; Me: methyl. Dashed lines mean uncharacterized regulation.

In plants, three mechanisms for redox regulation of DNA methylation may exist. One mechanism is redox components modulate the synthesis of methyl donor SAM. In plants, SAM synthesis is catalyzed by S-adenosylhomocysteine hydrolase (SAHH)/homologous gene silencing 1 (HOG1), methionine synthase (MS) and S-adenosyl methionine synthase (SAMS)/methionine adenosyltransferases (MAT) [69] (Figure 1). SAHH, MS and SAMS have been demonstrated to be S-nitrosated after treatment with NO donor S-nitrosoglutathione (GSNO) in *A. thaliana*. Activity assay showed that SAMS1 is reversibly inhibited by GSNO [70]. Also, proteomic studies have shown that many of the enzymes involved in SAM synthesis are targets of S-nitrosation and tyrosine nitration [71,72,73]; and the activity of SAHH is decreased by tyrosine nitration in sunflower [72].

In the SAM cycle, the precursor of SAM is methionine (Met), which is particularly susceptible to oxidation to methionine sulfoxide (MetSo) under stress conditions (Figure 1). Methionine sulfoxide reductases (MSRs) A and B catalyze the reduction of MetSo back to Met [74]. Accordingly, changes in the levels of NAD(H), NADP(H) and GRXs/thioredoxins (TRXs) may affect DNA methylation through controlling the concentrations of Met in cells. NAD(H) and NADP(H) can prevent Met oxidation. In *A. thaliana*, two MSR genes (*MSRB3* and *MSRB8*) are activated under high levels of NAD(H) and NADP(H), and accompanied with an increase in Met content [75]. GRXs/TRXs can donate electrons to MSRs for the catalysis of MetSo reduction [76]. These indicate that NAD(H), NADP(H), GRXs, and TRXs play important roles during the regeneration of Met; accordingly may modulate DNA methylation (Figure 1).

Additionally, accumulation of Met is dependent on the metabolism of folate, which provides 5-methyl-tetrahydrofolate (5-CH3-THF) for Met synthesis. In *A. thaliana*, impairment of folate production by sulfamethazine treatment has shown to reduce DNA methylation levels [77]. Folate metabolism can also contribute to the maintenance of redox balance by regulating NADPH production, further modulating DNA methylation in plants [78,79]. As shown in the folate cycle (Figure 1), dihydrofolate reductase-thymidylate synthase (DHFR-TS) is a bifunctional enzyme. Its subunit DHFR is located at the N terminus, and catalyzes the conversion of dihydrofolate (DHF) into tetrahydrofolate (THF) by consuming NADPH. THF and 5,10-CH_2_-THF can be interconverted by the enzyme serine hydroxymethyl transferase (SHMT). 5,10-CH_2_-THF can also be converted into DHF by TS. Methylenetetrahydrofolate dehydrogenase/methenyltetrahydrofolate cyclohydrolase1 (MTHFD1) is also a bifunctional enzyme, and can convert 5,10-CH_2_-THF into 5,10-CH=THF, leading to NADPH formation. Mutation in *MTHFD1* has been demonstrated to disturb folate metabolism and cellular redox state, and lead to loss of DNA methylation in *A. thaliana* [79]. In the *Arabidopsis* genome, three *DHFR-TS* genes exist. DHFR-TS3 inhibits DHFR-TS1 and DHFR-TS2. Overexpression of *DHFR-TS3* leads to decreases of DHFR and MTHFD activities, which in turn cause a drop of NADPH/NADP^+^ ratio [78], and likely impact DNA methylation. Further, 5,10-CH=THF is converted by methylenetetrahyrofolate reductase (MTHFR) to 5-CH3-THF, which enters the SAM cycle and serves for homocysteine (Hcy) remethylation to Met by MS.

The second mechanism for redox regulation of DNA methylation is that ROS and NO affect the expression and activities of DNA methyltransferases (DNMTs) and DNA demethylases. In *A. thaliana*, ROS mediate the irradiation-triggered DNA demethylation of bystander aerial plants. Irradiation of the roots markedly decreases the expression of *DRM2*, and enhances the transcriptional abundances of *MET1* and *DML3* in bystander aerial plants [80]. Similarly, application of SNP to rice plants induces DNA hypomethylation through down regulation of DNA methyltransferase genes *OsCMT2* and *OsCMT3* and upregulation of the DNA demethylase gene *OsDME* [67]. Yet, it is not clear whether the observed DNA hypomethylation in the SNP-treated plants are due to the regulation of DNA methylase activities or due to the NO-mediated post-translational modification of SAMS.

DNA glycosylases ROS1 and DME have DNA demethylase activity. They catalyze the excision of entire methylated cytosine instead of the methyl group through the base excision repair pathway. ROS1 and DME possess Fe-S cluster assembled structure as their cofactor, and the Fe–S binding motif is essential for their enzymatic activity [81]. Fe-S cluster can gain or lose electrons under different oxidation conditions [82]. Accordingly, redox components may modulate DNA demethylation through impacting the activity of Fe-S cluster assembled DNA demethylases like ROS1 and DEM in plants.

**Figure 2:**
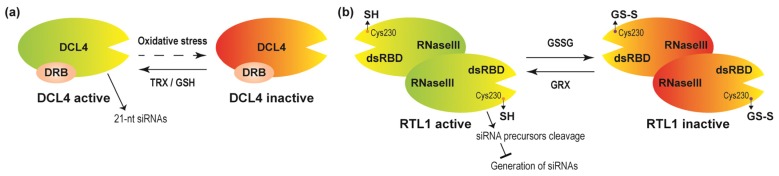
Redox components regulate DCL4 and RTL1 activities. (**a**) Processing of siRNA precursors by DCL4 requires dsRNA-binding protein (DRB), especially DRB4. GSH and TRXs are able to restore DCL4 activity from the inactive state. Activated DCL4 promotes 21 nt siRNA production. (**b**) GSSG/GRXs influence RTL1 activity. RTL1 has RNase III domain and dsRNA binding domains (dsRBD), and acts dimers to perform functions. GSSG treatment results in RTL1 glutathionylation at Cys230 position and inhibits its activity. RTL1 activity is restored by glutaredoxin proteins (GRXs). RTL1 negatively regulates siRNA production prior to DCL–mediated cleavage of the siRNA precursors. The dashed line indicates uncharacterized regulation.

The third mechanism for redox modulation of DNA methylation level is that redox mediators modify the activities of dicer-like4 (DCL4) and RNAse III-like 1 (RTL1), thus affecting the production of siRNAs likely required for DNA methylation through RdDM pathway in plants [83,84]. siRNAs originate from inter- or intramolecular double-stranded RNA (dsRNA) precursors, which are catalyzed by dsRNA-specific endoribonucleases, DCL proteins [85]. In *A. thaliana*, the products of DCL2, DCL3, and DCL4 are 22-, 24- and 21-nt siRNAs, respectively [27,85]. Apart from DCL-mediated dicing activities, RTL1 also influences siRNA production by cleaving the dsRNA before processing by the DCL proteins, and thus acts as a negative regulator of siRNA production [86]. The roles of DCL4 and RTL1 proteins in siRNA production are depicted in Figure 2. In *A. thaliana*, the activity of DCL4 is suppressed by sulfur deficiency. The DCL4 activity can be recovered by supplementation with GSH and TRXs. Moreover, immunopurified DCL4 can be activated by recombinant thioredoxin-h1 with dithiothreitol in vitro, suggesting that DCL4 is under redox regulation. Activation of DCL4 can promote 21-nt siRNA production, and may further promote DNA methylation [83] (Figure 2a). Additionally, Arabidopsis RTL1 has dsRNA binding domains (dsRBD), in which one conserved cysteine (Cys230 in Arabidopsis RTL1) exists. Cys230 has been demonstrated to be crucial for RTL1 cleavage activity. In the presence of GSSG, RTL1 can be glutathionylated at Cys230. Moreover, glutathionylation of RTL1 clearly inhibits its cleavage activity, and the activity of glutathionylated RTL1 can be recovered by two GRX members GRXC1 and GRXC2, indicating that RTL1 is redox regulated (Figure 2b). Moreover, RTL1 negatively regulates siRNA production prior to DCL–mediated cleavage of the siRNA precursors (Figure 2b) [84]. Thus, redox mediators modulate siRNA generation through influencing the activities of DCL4 and RTL1 in plants, and further affect the DNA methylation level.

### 4.2. Redox Adjustment of Histone Methylation

Similar to those of DNA methylation, the methyl groups of histone methylation are also derived from SAM. Thus, redox factors modulating SAM availability also modify histone methylation, as described in the DNA methylation section (Figure 1). In addition to influencing SAM synthesis, redox intermediates also regulate the expression and activity of HMTs and HDMs. For instance, application of S-nitrosocysteine, a NO donor, to *Arabidopsis* leaves upregulates the expression of *Set Domain Group 20*, a gene encoding lysine methyl transferase, and *PcG* *Histone Methyltransferase Curly Leaf* gene [87], pointing to the important function of NO in modulating the two HMTs. PRMT5 can catalyzes Arg symmetric dimethylation of histones and non-histone proteins in higher eukaryotes [88]. It has been reported that NO positively regulates PRMT5 activity by S-nitrosylation at Cys-125 under NaCl stress in *A. thaliana* [20]. Treatment with NO donor S-nitrosocysteine also prominently promotes JMJs expression in *A. thaliana* [87], implying that JMJs are possibly regulated by NO.

### 4.3. Redox Regulation of Histone Acetylation

**Figure 3:**
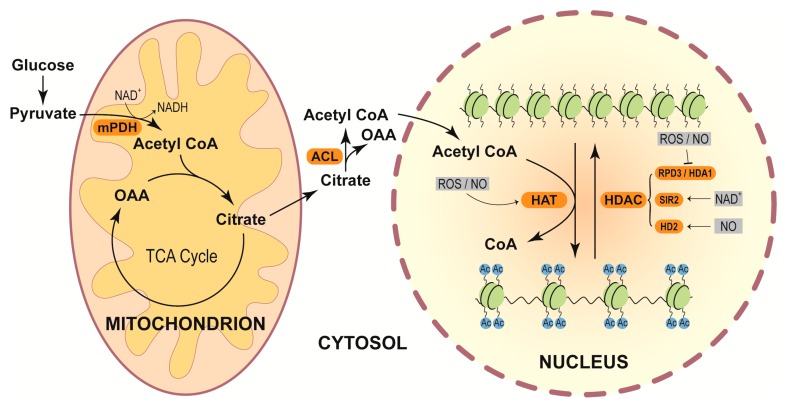
Redox components influence histone acetylations. In the cytoplasm, glucose is broken down to pyruvate, which enters into mitochondria, and is converted to acetyl CoA through mitochondrial pyruvate dehydrogenase (mPDH) by reducing NAD^+^. Acetyl CoA combines with oxaloacetate (OAA) produced in the TCA cycle to form citrate, which enters cytoplasm. In cytoplasm, citrate is converted back to OAA and acetyl CoA through ATP-citrate lyase (ACL). Acetyl CoA synthesized in the cytoplasm enters into the nucleus as the source supplier of acetyl group for the histone acetylation process. HAT utilizes the acetyl group from acetyl CoA to introduce acetylation marks (Ac) over the lysine residues of the histone tail, thus weakening the contact between DNA and histone and facilitating gene expression. HDAC removes histone acetyl group, leading to chromatin compaction. Different HAT and HDAC enzymes are affected by ROS, NO, and NAD^+^.

Increasing evidence suggests that redox components regulate histone acetylation through affecting acetyl CoA accumulation. It has been addressed that pyruvate conversion to acetyl CoA is catalyzed by pyruvate dehydrogenase (PDH) complex, which uses NAD^+^ as a cofactor for its catalytic activity (Figure 3). Increases in the ratio of NADH to NAD^+^ in *Escherchia* *coli* inhibit PDH activity, and block the acetyl CoA formation [89]. An in vitro study also revealed that elevation in ratio of NADH/NAD^+^ is associated with the inhibition of PDH activity in pea [90]. Yet, whether the inhibited PDH activity causes the decreases in levels of acetyl CoA in plants remains to be determined.

Changes in redox reagents also modulate the activities of HATs and HADCs in plants (Figure 3). It has been documented that heat stress promotes the accumulation of O_2_^•−^ and induces PCD, followed by histone hyperacetylation due to the elevated expression of genes *HAT-B* and *General Control Nondepressible 5 (GCN5)* in maize seedlings [91]. Dietzel et al. [92] detected the early nuclear target genes of plastidial redox signals in responding to a reduced light-induced signal of the photosynthetic electron transport chain in *A. thaliana*, and found that many nuclear genes are not expressed in the redox compromised state transition 7 (stn7) mutants but expressed in WT. Among these, several are epigenetically regulated. Further studies revealed that the redox signal from chloroplasts of WT rather than stn7 activates the nuclear HAT and HDAC, which promote histone acetylation and deacetylation, respectively [92]. Similarly, *Arabidopsis* mutants accumulated high levels of H_2_O_2_ (*cat2*) and were defective in GSSG to GSH conversion (*gr1*), showing the differential expression of GCN5-related acetyl transferase gene [93].

In *A. thaliana*, the expression of many pathogensis-related (PR) genes is suppressed by HDA19, which deacetylates the histones on PR protein promoters under nonpathogenic condition. Pathogen attack abolishes HDA19 activity, further resulting in the acetylation of PR protein promoters and increased expression of PR-related genes [94]. Pathogen infection induces oxidative burst at a very early time [95]. Thus, the HDA19 activity affected by pathogen attack is most likely regulated by ROS. Indeed, Liu et al. [96] found that salicylic acid (SA) and flagellin 22 (a bacterial protein) trigger ROS production, leading to the oxidation of HDA9 and HDA19 in *A. thaliana*. The oxidation of the HDACs reduces their activity and further increases the histone acetylation of stress-responsive genes.

In plants, the activities of sirtuin HDACs are dependent on the NAD^+^ level and NAD^+^/NADH ratio [21]. Thus, oxidative stress alters the redox status of NAD^+^, and may further imprint on HDAC activity. In rice, NAD^+^-dependent sirtuin OsSRT1 has been reported to play critical roles in suppressing glycolysis by deacetylating histones and glyceraldehyde-3-phosphatedehydrogenase [97]. Redox mediators likely modulate the catalytic activity of OsSRT1 by affecting NAD^+^ level.

Mengel et al. [98] found that NO donors GSNO and S-nitroso-N- acetyl-DL-penicillamine and glutathionylating reagent GSSG reversibly suppress HDAC activity in *A. thaliana*. S-nitrosylation has stronger effects than S-glutathionylation on the HDAC activity. In addition, SA has been shown to induce endogenous NO generation, which represses HDAC activity and stimulates histone acetylation [98]. Moreover, GSNO or GSH clearly increases whereas NO scavenger 2-(4-carboxyphenyl)-4,4,5,5-tetramethylimidazoline-1-oxyl-3-oxide decreases acetylation levels of many H3K9/14ac sites, indicating that NO contributes to the GSNO-triggered hyperacetylation. HD2 proteins are plant-specific HDACs. The expression of HD2-like gene *DlHD2* and two ethylene-responsive factor-like genes *DlERF1* and *DlERF2* enhances during longan fruit senescence. Treatment with NO delays the fruit senescence, elevates the transcription of *DlHD2*, but diminishes the expression of *DlERF1* and *DlERF2*. These data imply that NO modulates fruit senescence possibly through affecting the expression of *HD* gene in longan [99]. Additionally, in *A. thaliana*, the HD2 type member HDT2 (histone deacetylase 2) and HDT3 have been identified to be S-nitrosylated [100].

### 4.4. Redox Affecting Chromatin Remodelers and Other Chromatin-Associated Factors

DNA methylation 1 (DDM1) is an important SWI/SNF2 chromatin remodeler, and can shift nucleosome composition and mediate DNA methylation by allowing MET1, CMT2, and CMT3 to access DNA, especially in heterochromatin regions in plants [101]. Mutation of DDM1 leads to a dramatic decrease in DNA methylation in *A. thaliana* [102]. In rice, exogenous application of SNP results in the downregulation of the expression of *OsDDM1a* and *OsDDM1b*, as well as DNA hypomethylation [67], indicating that NO possibly modulates DNA methylation via impacting chromatin remodeling. PICKLE, a CHD3 remodeler, promotes H3K27me3 in *A. thaliana* [103]. It is identified as a target for tyrosine nitration [73], suggesting that its activity is redox regulated. In *A. thaliana*, topoisomerase VI (Topo VI) A subunit (AtTOP6A), a chromatin-associated factor, has been demonstrated to mediate singlet oxygen signals from the plastid to the nucleus. Under ^1^O_2_ accumulation condition, AtTOP6A binds to the promoters of ^1^O_2_-responsive *AAA-ATPase* gene and a set of other ^1^O_2_-responsive genes, and directly activates the expression of these genes. Topo VI also regulates the transcription of H_2_O_2_-responsive genes under high light stress. However, changes in the expression of ^1^O_2_- and H_2_O_2_-responsive genes modulated by AtTOP6A are different, suggesting that Topo VI is capable of integrating multiple signals produced by ROS in plants under stress [104].

## 5. Conclusions

Redox mediators, particularly ROS and NO have been emerging key regulators of chromatin remodeling in plants. They greatly influence not only the transcription and activities of multiple enzymes, catalyzing the addition or removal of methyl and acetyl groups in DNA and histones, but also the biosynthesis and supply of methyl and acetyl donors to DNA and histones. Redox-regulated changes in the epigenetic marks shape chromatin organization, further controlling the expression of many genes and other molecular processes, thereby profoundly affecting plant growth and stress responses. In recent years, much progress has been made on the roles of redox mediators in regulating DNA methylation, and histone modifications in plants. However, many reported actions of redox components on epigenetic marks are indirect effects, and the precise molecular mechanisms underlying the processes are largely unknown. Whether epigenetic modification changes are caused by one oxidant without triggering other antioxidants is also poorly described. Moreover, the research works on redox regulation of other epigenetic marks like phosphorylation, ubiquitinylation, glycosylation, ADP-ribosylation, and sumoylation of histone, chromatin remodeling, and siRNA are quite limited to date.

It has been documented that pathogen attack and diverse abiotic stresses significantly modify epigenetic marks [6,8,9]. ROS, NO and other redox components are also central mediators of these environmental stresses [16,18]. Yet whether the stress triggered chromatin modifications are dominantly mediated by the redox intermediates remains to be determined. Additionally, NADPH oxidase, and multiple antioxidant enzymes contribute to ROS generation and scavenging, respectively, and nitrate reductase and NO synthase-like enzymes are responsible for NO biosynthesis in plants [18,47,54,55]. However, whether these enzymes play important roles in epigenetic modifications is unclear. We believe that these problems will be solved in near future with the rapid development of various biotechnologies, including omics, bioinformatics, and gene editing technologies. We also believe that uncovering the molecular mechanisms for redox control of epigenetic changes will greatly help to understand the strategies of plants adapting to ever-changing environmental conditions, and to facilitate cultivating of elite crop varieties with desired characteristics in the coming days.

## Abbreviations

ACL	ATP-citrate lyase
APX	Ascorbate peroxidase
ASC	Ascorbate
CAT	Catalase
CHD	Chromodomain helicase DNA-binding
CMT	Chromomethylase
DCL	Dicer-like
DDM1	DNA methylation 1
DHF	Dihydrofolate
DHFR	Dihydrofolate reductase
DME	Demeter
DML	Demeter-like
DNMT	DNA methyltransferase
DRB	dsRNA-binding protein
DRM	Domain rearranged methyltransferase
dsRBD	dsRNA binding domains
GCN5	General Control Nondepressible 5
GPX	Glutathione peroxidase
GR	Glutathione reductase
GRX	Glutaredoxin
GSH	Glutathione
GSNO	S-Nitrosoglutathione
GSSG	Oxidized glutathione
H_2_O_2_	Hydrogen peroxide
HAT	Histone acetyltransferase
Hcy	Homocysteine
HD2	Histone deacetylase 2
HDAC	Histone deacetylase
HDM	Histone demethylase
HKMT	Histone lysine methyltransferase
HMT	Histone methyltransferase
HDT2	Histone deacetylase 2
JMJ	Jumonji C domain-containing protein
LSD1	Lysine-specific demethylase 1
Met	Methionine
MET1	DNA methyltransferase 1
MetSo	Methionine sulfoxide
MS	Methionine synthase
MSR	Methionine sulfoxide reductase
MTHFD1	Methylenetetrahydrofolate dehydrogenase/methenyltetrahydrofolate cyclohydrolase1
MTHFR	Methylenetetrahyrofolate reductase
NAD(P)H	Pyridine nucleotides
NO	Nitric oxide
OAA	Oxaloacetate
^1^O_2_	Singlet oxygen
O_2_^•−^	Superoxide radical
PCD	Programmed cell death
PDH	Pyruvate dehydrogenase
PR	Pathogensis-related
PRMT	Protein arginine methyltransferase
RBOH	Respiratory burst oxidase
RdDM	RNA directed DNA methylation
RDP3/HDA1	Reduced potassium dependency 3/histone deacetylase 1
ROS	Reactive oxygen species
ROS1	Repressor of silencing 1
RTL	RNAse III-like
SA	Salicylic acid
SAM	S-Adenosyl-L-methionine
SAMS/MAT	S-Adenosyl methionine synthase/methionine adenosyl transferases
SAHH/HOG1	S-Adenosylhomocysteine hydrolase/homologous gene gilencing 1
SHMT	Serine hydroxylmethyl transferase
siRNA	Small interfering RNA
SIR2	Silent information regulator 2
SNP	Sodium nitroprusside
SOD	Superoxide dismutase
stn7	State transition 7
SWI/SNF	Switching defective/sucrose non-fermenting
THF	Tetrahydrofolate
Topo VI	Topoisomerase VI
TRX	Thioredoxin
TS	Thymidylate synthase
